# The longitudinal trajectories of mental health outcomes in healthcare workers in England during the COVID-19 pandemic

**DOI:** 10.1017/S0033291726104280

**Published:** 2026-05-14

**Authors:** Chris Penfold, Pamela Almeida-Meza, Theresa Redaniel, Lauren J. Scott, Paul Moran, Rosalind Raine, Rupa Bhundia, Neil Greenberg, Simon Wessely, Bethany Croak, Richard Morriss, Ira Madan, Peter Aitken, Anne Marie Rafferty, Sarah Dorrington, Dominic Murphy, Sharon A.M. Stevelink, Danielle Lamb

**Affiliations:** 1 Population Health Sciences, Bristol Medical School, University of Bristol, UK; 2 Institute of Psychiatry, Psychology & Neuroscience, King’s College London, UK; 3 Department of Primary Care and Population Health, University College London, UK; 4 NIHR ARC East Midlands, University of Nottingham, UK; 5 Guy’s and St Thomas’ NHS Trust, UK; 6 Sussex Partnership NHS Foundation Trust, UK; 7 Florence Nightingale Faculty of Nursing, Midwifery & Palliative Care, King’s College London, UK

**Keywords:** COVID-19, Healthcare workers, Mental health, Longitudinal study, Trajectory analysis, Growth mixture modelling, Anxiety, Depression, PTSD, Occupational health, NHS staff, Workplace support

## Abstract

**Background:**

The COVID-19 pandemic raised concerns about the mental health of an already burdened healthcare workforce. This study examined mental health trajectories among healthcare workers (HCWs) across the pandemic and identified personal and employment factors associated with different symptom patterns.

**Methods:**

Longitudinal data were drawn from the NHS CHECK cohort, including clinical and non-clinical staff from 18 NHS Trusts in England (April 2020–April 2023). Growth curve and growth mixture models identified latent classes of HCWs characterized by distinct trajectories of probable common mental disorders. Secondary outcomes included anxiety, depression, alcohol misuse, and post-traumatic stress symptoms. Logistic regression examined associations between baseline personal and employment characteristics and class membership.

**Results:**

The analytical sample included 22,764 participants. For each outcome, growth mixture models identified two latent classes. Approximately 31% of HCWs experienced persistently high symptoms of probable common mental disorders, while 69% experienced persistently low symptoms. Similar patterns were observed for secondary outcomes, with small subgroups demonstrating worsening symptoms followed by improvement. Logistic regression analyses showed that being female, younger, single, working as a nurse, or having a pre-existing mental health diagnosis increased the odds of belonging to a high symptom class. Perceived support from colleagues and managers was protective.

**Conclusions:**

While many HCWs reported consistently low mental health symptom levels, almost a third belonged to a latent class characterized by persistently high symptoms across all time points. These findings underscore the need for mental health support for vulnerable HCW groups, embedded within routine NHS practice rather than limited to crisis periods.

## Introduction

The COVID-19 pandemic raised concerns about the mental health of healthcare workers (HCWs), a workforce that was already experiencing high levels of occupational stress prior to the pandemic (Almeida-Meza, Ledden et al., 2025; Søvold et al., 2021). Understanding how the mental health of HCWs has changed over time, and the factors influencing these changes, is critical for informing support strategies and safeguarding workforce sustainability.

Throughout the pandemic, international and UK studies reported high prevalence of anxiety, depression, and Post-Traumatic Stress Disorder (PTSD) among HCWs, often exceeding rates seen in the general population both before and during the COVID-19 pandemic. For example, pooled prevalence estimates from systematic reviews and meta-analyses have ranged from 24% to 68% for anxiety, 12% to 56% depression, and 20% to 31% for PTSD (Huang et al., 2024; Maqbali et al., 2024; Marvaldi et al., 2021; Vizheh et al., 2020). These estimates are in line with levels of mental health symptoms found in HCWs in previous health emergencies (Kisely et al., 2020; Preti et al., 2020) and are higher than the estimates from the general population before (4.4% anxiety and 3.6% depression) and during (27% for anxiety, 28% for depression, and 24% for PTSD) the COVID-19 pandemic (Nochaiwong et al., 2021; WHO, 2017).

In the UK, even prior to COVID-19, HCWs were already at increased risk of mental health problems, with studies reporting prevalence ranging from 27% to 48% for anxiety, 7% to 38% for depression, and 8% to 14% for PTSD, depending on medical specialty (Imo, 2017; Kinman & Teoh, 2018). While it might be expected that frontline HCWs would experience more substantial mental health problems due to the nature of their work, there is conflicting evidence about this (Pierce et al., 2020). To date, much research has considered only clinical staff, limiting generalizability. Furthermore, nearly all previous studies finding high levels of poor mental health among HCWs have relied on data from the first wave of the pandemic, cross-sectional data, limited sample sizes, over-reliance on convenience samples, or data with a short duration of follow-up (Greenberg et al., 2021; Manchia et al., 2022).

The NHS CHECK study aimed to address these evidence gaps by including a substantial sample of nationwide HCWs in 18 participating Trusts (clinical and non-clinical) and providing longitudinal data collected from near the start of the pandemic (April 2020) until May 2023. The study addressed the following research questions:

1. What was the average trajectory of mental health symptoms among HCWs across the COVID-19 pandemic?
2. Were there latent subgroups of HCWs characterized by different symptom trajectories over time?
3. Which personal (e.g. age, sex, relationship status, pre-existing mental health conditions) and organizational factors (e.g. role, perceived support) are associated with membership to higher-symptom trajectories?

We hypothesized that most HCWs would experience elevated mental health symptoms early in the pandemic, with symptoms subsiding over time. We further hypothesized the presence of distinct subgroups, including one characterized by persistently worse or more prolonged symptoms. This higher-symptom subgroup was expected to comprise younger, female, and single individuals, those with a pre-existing mental health condition, those in lower occupational roles, and those reporting lower perceived support from colleagues and managers.

## Methods

### Study design and population

We used a longitudinal cohort study design to investigate the trajectories of mental health symptoms among HCWs. Latent growth curve models (LGCMs) described the average trajectory of mental health symptoms, and growth mixture models (GMMs) identified subpopulations of HCWs who follow different symptom trajectories over time (Herle et al., 2020).

We included respondents to the NHS CHECK cohort as defined in the study protocol (Lamb et al., 2021). In summary, all staff working at 18 participating NHS Trusts across England were invited to participate. The baseline data collection was from April 2020 to January 2021. Follow-up data were collected approximately 6 months (data collection closed on August 2021), 12 months (data collection closed on February 2022 – including the replenishment cohort who joined at this stage), and 32 months (data collection closed on May 2023) after their baseline. The replenishment cohort was comprised of HCWs from participating NHS Trusts who had not taken part at baseline but completed the same survey instruments at the 12-month wave. They were included to mitigate attrition and maintain representativeness over time.

Participants were only included in this analysis if they had completed the baseline questionnaire and had complete sex, ethnicity, age, role, and Trust data to be weighted in the dataset (Lamb et al., 2021; see Supplementary Table 1).

Ethical approval for the NHS CHECK study was granted by the Health Research Authority (reference: 20/HRA/2107, IRAS: 282686) and local Trust Research and Development approval. Informed consent was collected electronically prior to participation.

#### Outcome of interest

All outcomes were assessed at baseline (April 2020–January 2021) and approximately 6, 12, and 32 months later, using the same measures at each wave.

Our primary outcome was the prevalence of probable common mental disorders measured using the General Health Questionnaire (GHQ-12) (Goldberg & Hillier, 1979). This was included as the total score (0–12) to model the average trajectories and dichotomized for temporal trends analysis, with scores of four and above indicating probable mental disorder. There was a small typographical error in one response option to the GHQ-12 (baseline, 6 months, and 12 months). Separately, we tested the impact of this error through an RCT on overall GHQ score and found scores were unaffected by this error (Croak et al., 2024).

Our secondary outcomes included measures of anxiety, depression, problem drinking, and PTSD. Anxiety was assessed using the Generalized Anxiety Disorder scale (GAD-7) (Spitzer, Kroenke, Williams, & Lowe, 2006), with a total scale ranging from 0 to 21. Scores of 10 or above were considered indicative of symptomatology consistent with anxiety disorder (caseness). Depression was measured using the Patient Health Questionnaire (PHQ-9) (Kroenke, Spitzer, & Williams, 2001), with a total score ranging from 0 to 27. Scores of 10 or above indicated caseness for probable depression. Problem drinking was evaluated using the Alcohol Use Disorder Identification Test (AUDIT-C) (Babor, Higgins-Biddle, Saunders, & Monteiro, 2001), with a total score ranging from 0 to 45. Scores of 8 or above were considered indicative of problematic drinking. PTSD symptomatology was assessed using the Post-Traumatic Stress Disorder checklist (PCL-6) (Lang & Stein, 2005), with a total score ranging from 6 to 30. Scores of 14 or above were indicative of probable caseness for PTSD. The scores of each of these scales were included as continuous and dichotomized (indicator of caseness) variables.

#### Independent variables

Based on the literature (Almeida-Meza, Ledden et al., 2025; Carr et al., 2022; Jordan et al., 2023; Pierce et al., 2021), we examined personal and employment factors that have been associated with mental health outcomes amongst HCWs. Personal factors included sex (female or male), age at baseline survey (≤30, 31–40, 41–50, 51–60, and ≥61 years), ethnicity (African/Caribbean/Black British, Asian/Asian British, Mixed/Multiple ethnic groups, other racial and ethnic minority groups and White), marital status (categorized as ‘in a relationship’, including married, in a civil partnership, cohabiting, or in a relationship, and ‘single’, including divorced, separated, widowed, or single), clinical role (doctor, nurse, other clinical, or non-clinical), and history of pre-existing depression or PTSD (yes or no).

Employment factors included trust type (Acute or Mental Health), whether the individual had been redeployed outside their usual role (yes or no), and perceived support from colleagues and managers (yes or no).

Additionally, COVID-19 case numbers were retrieved from NHS England and matched to the trust locality and time of data collection (UK Health Security Agency, 2023).

### Statistical analyses

#### Descriptive data

Continuous variables were summarized using means and standard deviations (SD) (or medians and interquartile ranges (IQR) if the distribution is highly skewed), while categorical data were summarized as numbers and percentages.

#### Data analysis

We described unweighted and survey-weighted mental health outcomes at each time point as both i) continuous outcomes and ii) caseness. We described within-person change in continuous mental health outcomes using GMM. Continuous mental health scale scores were modeled as repeated outcomes defining the latent trajectories. We developed the GMMs in two main stages:

1. We first fitted LGCMs to identify the most appropriate functional form of growth. In the absence of literature to guide the selection of functional form, we compared linear models with linear spline models using two and three knots. Knots (the points where the growth pattern could change) were evenly distributed across the data collection time periods, meaning they were placed at regular intervals across the study timeline.
2. We then fitted GMMs with increasing numbers of latent classes. The optimal number of classes was selected based on relative model fit and interpretability. Relative model fit was assessed using the Akaike information criterion (AIC) and the sample size adjusted Bayesian information criterion (saBIC). Lower AIC and saBIC indicate better fit (Carr et al., 2022). Models were compared across functional forms and 1–5 class solutions.

Time was incorporated in the development of the LGCMs as the date of questionnaire completion, and time was centered at the earliest date of completion of the baseline questionnaires. The daily COVID-19 case numbers for each NHS region (UK Health Security Agency, 2023) were linked with questionnaire responses by date and Trust and were treated as a time-varying predictor of class membership.

We explored associations between baseline covariates and latent class membership using a three-step approach (Kamata, Kara, Patarapichayatham, & Lan, 2018). The covariates were analyzed in the auxiliary model and were not included in the GMM used to derive trajectories.

1. Derive the GMM as above (maximum iterations = 500, no random starts).
2. Derive a variable for the most likely class and retain its associated measurement error (classification uncertainty rate derived from estimated posterior class probabilities and number assigned to each class).
3. Fit the auxiliary model separately for each class by incorporating the measurement error from step 2.

We followed the Guidelines for Reporting Latent Trajectory Studies (GRoLTS) checklist (van de Schoot et al., 2017; see Supplementary Table 2). Logistic regressions were used to estimate the odds of class assignment. For all outcomes, the class with the lowest scores over the study was the reference class.

#### Model assumptions and missing data

LGCMs allow for an unbalanced design, meaning the measurement of outcome is not necessary for every time point. A participant who contributed fewer than the maximum number of observations can be retained in the model. However, missingness is assumed missing at random. We compared the baseline characteristics and outcome scores between people with complete and incomplete 32-month GHQ-12 scores to explore systematic variation in who responded (see Supplementary Table 3).

#### Statistical software

Analyses were performed in R version 4.2 using the ‘lcmm’ package (Proust-Lima, Philipps, & Liquet, 2015). All statistical code is available at DOI: https://doi.org/10.5281/zenodo.18682400

## Results

### Study sample and GHQ-12 response rates at each survey point

The baseline sample for NHS CHECK was 22,764 participants (see Supplementary Table 1). Baseline response rates for GHQ-12 were 86.8%, 40.4% at 6 months, 47.6% at 12 months, and 27.8% at 32 months. See Supplementary Table 4 for the mean and cut-off scores for the mental health outcomes at all timepoints. Additionally, the date ranges for questionnaire completion at each time point are shown in Supplementary Figure 1.

### Cohort characteristics

The baseline sociodemographic and workplace characteristics are described in Table 1. The sample comprised 22,764 participants, of which 21,236 (93.0%) of eligible participants had completed the baseline questionnaire, and 1,528 were recruited at 12-months follow up as a replenishment cohort. The sample was predominantly female (76.0%) and largely composed of younger and middle-aged individuals, with 49.0% falling within the 31 to 50 age range. In terms of ethnicity, the participants were primarily White (76.0%), with a smaller proportion of participants from Asian (12.0%), Black (8.1%), and other minority groups. Acute trusts accounted for 71.0% of participants, of which most held clinical roles, including nurses (30.0%) and other clinical staff (33.0%). Nearly three-quarters of the sample were in a relationship, including married, civil partnership, or cohabiting. Most of the sample reported feeling supported by colleagues (75.0%) and managers (62.0%). Furthermore, a small proportion of participants reported having been redeployed (12.0%) and having pre-existing diagnoses of PTSD (1.3%) or depression (8.2%).

**Table 1.** Characteristics of participants at baseline

Characteristic	Count	Unweighted proportion	Weighted proportion
	*N* = 22,764	*N* = 22,764	
**Cohort**			
Main cohort	**21,236**	**93.0%**	**94.0%**
Replenishment cohort	**1,528**	**6.7%**	**6.1%**
**Sex**			
Female	18,469	81.0%	76.0%
Male	4,295	19.0%	24.0%
**Age, years**			
≤30	4,614	20.0%	22.0%
31–40	5,187	23.0%	26.0%
41–50	5,938	26.0%	23.0%
51–60	5,612	25.0%	22.0%
≥61	1,413	6.2%	6.8%
**Ethnicity**			
Asian/Asian British	1,479	6.5%	12.0%
Black/African/Caribbean/Black British	981	4.3%	8.1%
Mixed/Multiple racial and ethnic groups	570	2.5%	1.1%
Other racial and ethnic minority groups	203	0.9%	2.2%
White	19,531	86.0%	76.0%
**Main role**			
Doctor	1,649	7.2%	9.8%
Nurse	5,869	26.0%	30.0%
Other clinical	7,047	31.0%	33.0%
Non-clinical	8,199	36.0%	27.0%
**Marital status**			
Married/Civil partnership	11,048	49.0%	49.0%
Co-habiting/In a relationship	5,695	25.0%	24.0%
Divorced/separated/widowed	1,728	7.6%	6.9%
Single	4,243	19.0%	20.0%
Missing data	50		
**Type of trust**			
Acute	11,257	49.0%	71.0%
Mental health	11,507	51.0%	29.0%
**Redeployed**			
No (usual role)	18,444	87.0%	88.0%
Yes	2,636	13.0%	12.0%
Missing data	1,684		
**Felt supported by colleagues**			
Not at all/moderately	4,873	24.0%	25.0%
Yes	15,762	76.0%	75.0%
Missing data	2,129		
**Felt supported by manager**			
Not at all/moderately	7,154	35.0%	38.0%
Yes	13,462	65.0%	62.0%
Missing data	2,148		
**Pre-existing diagnosis of PTSD**			
No	20,971	99.0%	99.0%
Yes	265	1.2%	1.3%
Missing data	1,528		
**Pre-existing diagnosis of depression**			
No	19,367	91.0%	92.0%
Yes	1,869	8.8%	8.2%
Missing data	1,528		

People with complete 32-month GHQ-12 scores were older, from white ethnic groups, in non-clinical roles, and more likely to be either married/cohabiting or divorced/separated/widowed than those with incomplete 32-month GHQ-12 scores. There was no variation in other baseline characteristics, and baseline mental health scores were comparable for those with complete and incomplete 32-month GHQ-12 scores (see Supplementary Table 3).

### Trajectories of mental health outcomes

#### Results from latent growth curve models

**Figure 1.**
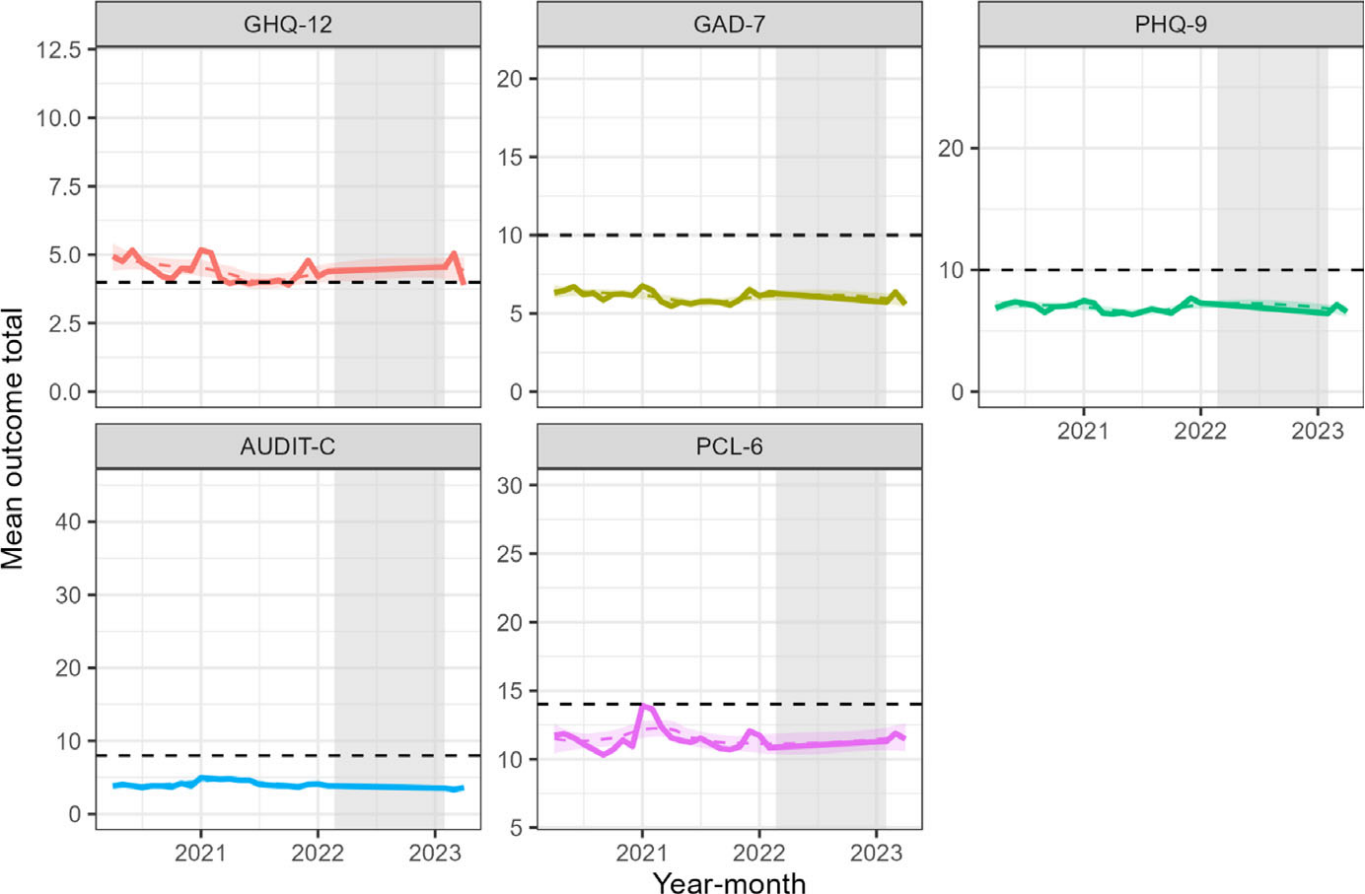
Temporal trends in monthly probable mental disorders (GHQ-12), anxiety (GAD-7), depression (PHQ-9), problematic drinking (AUDIT), and post-traumatic stress disorder (PCL-6) mean total scores. The gray shaded area represents the period of no data collection; the y-axis limits are set to the minimum and maximum possible values of each outcome. Dashed black lines are the cut-off for caseness for each mental health outcome; color-matched dashed lines and shaded areas are the Loess smoothed trend lines and 95% confidence intervals.

On visual inspection of the mental health outcomes over the study period, the mean prevalence of probable common mental disorders (GHQ-12) reduced modestly during 2020 and 2021 and remained relatively unchanged until 2023 (Figure 1). The mean total probable anxiety (GAD-7) and depression (PHQ-9) scores changed minimally over the study period. Mean problematic drinking (AUDIT-C) scores increased modestly at the end of 2020 and decreased throughout 2021; mean scores in 2023 were very similar. Probable PTSD (PCL-6) scores increased sharply at the end of 2020 and reduced to 2020 levels by summer 2021.

**Figure 2.**
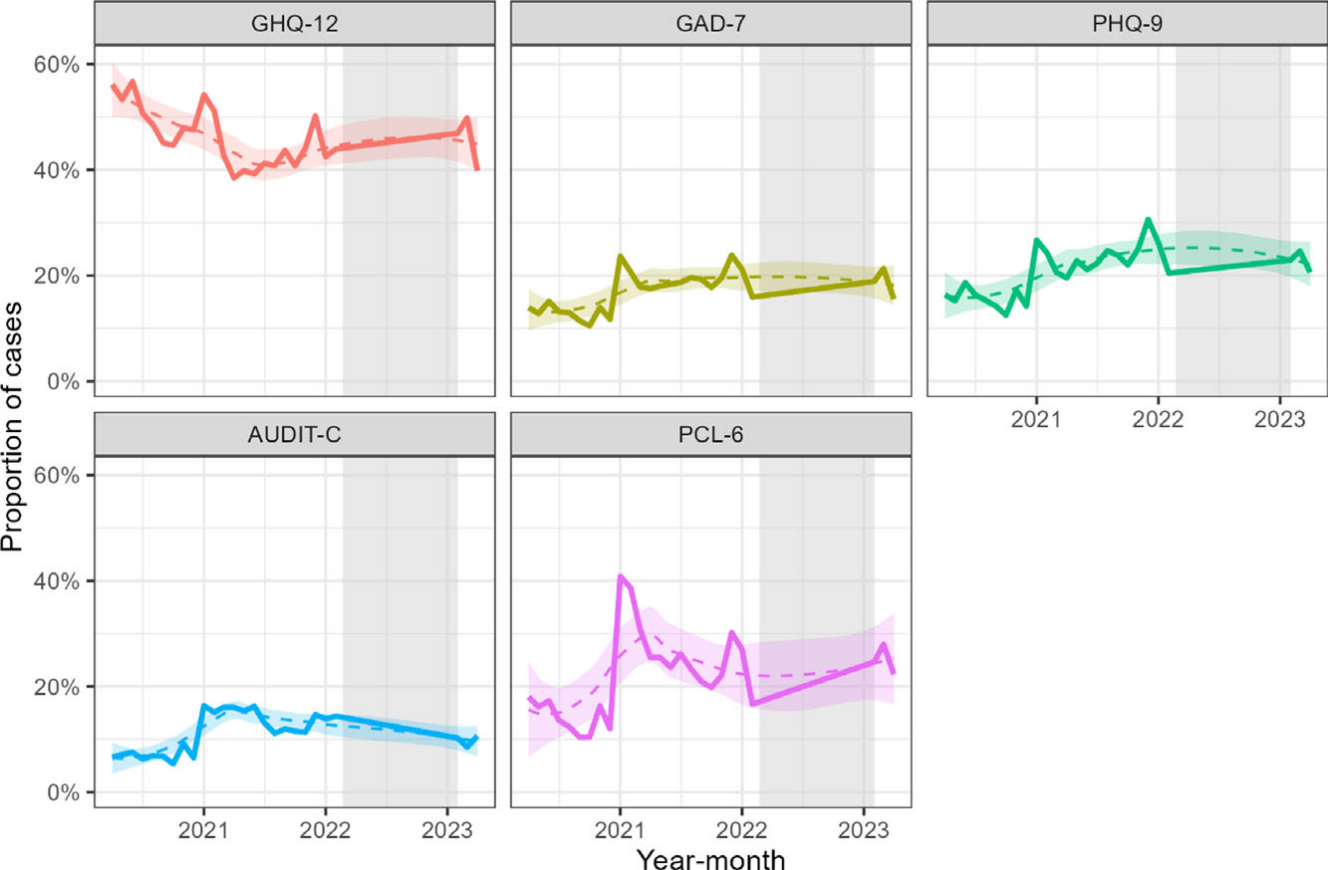
Temporal trends in the proportion of respondents per month classified as cases for probable mental disorders (GHQ-12), anxiety (GAD-7), depression (PHQ-9), problematic drinking (AUDIT), and post-traumatic stress disorder (PCL-6). The gray shaded area represents the period of no data collection; color-matched dashed lines and shaded areas are the Loess smoothed trend lines and 95% confidence intervals.

Figure 2 presents the temporal trends in the monthly proportion of cases for each outcome. The proportion of probable common mental disorder (GHQ-12) cases reduced during 2020 and 2021 to a minimum of 40%, and increased modestly until 2023, in which ~50% respondents were classed as cases. For probable anxiety (GAD-7), there was a small rise in the proportion of cases during 2020 and 2021, reaching a maximum of approximately 20% at the end of 2021, which changed minimally by 2023. The proportion of cases for probable depression (PHQ-9) increased from <20% in 2020 to ~25% in late 2021/early 2022. The proportion was very similar in 2023. The scores from problematic drinking (AUDIT) suggest that the proportion of cases increased sharply at the end of 2020 and similarly reduced in summer 2021, remaining similar in 2023. For PTSD (PCL-6), the proportion of cases rose sharply at the end of 2020 and declined throughout 2021. There was a further spike at the end of 2021, which declined rapidly in 2022 and was comparable in 2023.

#### Results from growth mixture models

**Figure 3.**
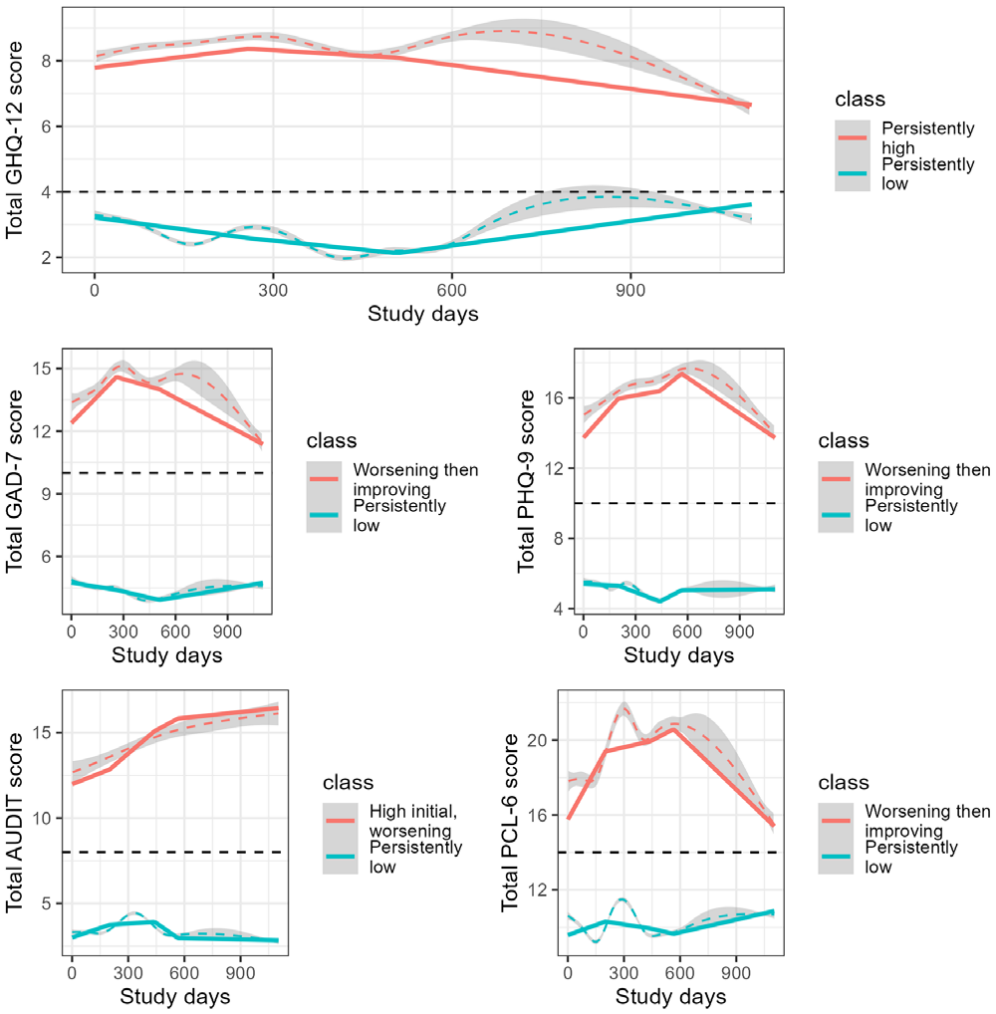
Predicted (solid line) and observed (dashed line) trajectories of probable mental disorders (GHQ-12), anxiety (GAD-7), depression (PHQ-9), problematic drinking (AUDIT), and post-traumatic stress disorder (PCL-6) scores by latent classes derived from the optimal GMMs. Dashed colored lines are the trajectories of overall mental health scores. Dashed black lines are the cut-off for caseness for each mental health outcome.

The trajectories of mental health scores are presented in Figure 3, with the descriptive labels we applied to the classes. For the main outcome, prevalence of probable common mental disorders (GHQ-12), a two-class solution was the optimal fit (Supplementary Table 5). Class 1: ‘Persistently high’ (31.0%) reported mean scores above the threshold for a probable mental disorder, which stayed high throughout. Class 2: ‘Persistently low’ (69.0%) reported mean scores below the threshold for probable mental disorder at the start of data collection, but scores were increasing after 500 days.

For all our secondary outcomes, a two-class solution was also the optimal fit (Supplementary Tables 6–9). GAD-7 Class 1: ‘Worsening then improving’ (17.1%) reported mean scores above the threshold for a generalized anxiety disorder over the entire study. Scores worsened over the first six months and returned to baseline levels by the end of the study. GAD-7 Class 2: ‘Persistently low’ (82.9%) had consistently low mean scores over the entire study.

PHQ-9 Class 1: ‘Worsening then improving’ (15.6%) reported mean scores above the threshold for depression caseness over the entire study. Scores worsened over the first six months and returned to baseline levels by the end of the study. PHQ-9 Class 2: ‘Persistently low’ (84.4%) had consistently low mean scores over the entire study.

AUDIT Class 1: ‘High initial, worsening’ (5.5%) had mean scores above the threshold for harmful or hazardous drinking at the start of the study, and their group mean scores worsened throughout the study. AUDIT Class 2: ‘Persistently low’ (94.5%) had consistently low mean scores over the entire study.

PCL-6 Class 1: ‘Worsening then improving’ (13.8%) had mean scores which were above the threshold for probable PTSD. Their mean score rapidly increased over the first year, followed by a period of improving scores. PCL-6 Class 2: ‘Persistently low’ (86.2%) had high mean scores below the threshold for probable PTSD throughout the study.

The model-estimated class-specific mean scores for the final two-class solutions are presented in Supplementary Table 10.

### Baseline predictors of trajectory class membership

#### Results from logistic regressions examining person and employment factors associated with common mental disorders trajectories

**Figure 4.**
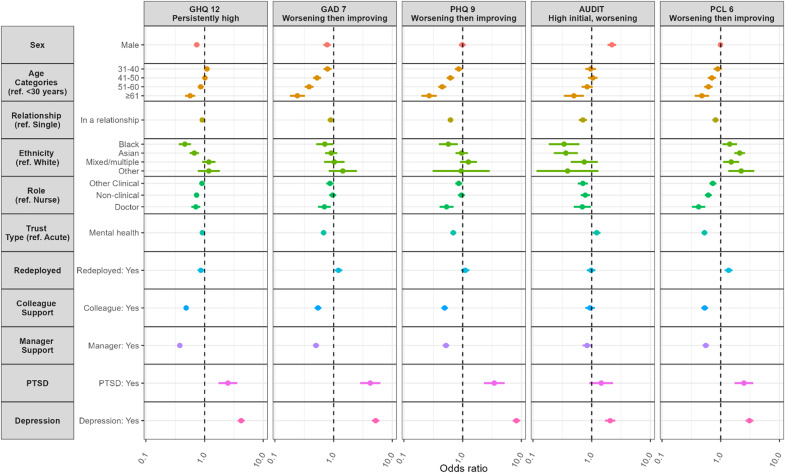
Adjusted associations between person and workplace factors and trajectories of probable mental disorders (GHQ-12), anxiety (GAD-7), depression (PHQ-9), problematic drinking (AUDIT), and post-traumatic stress disorder (PCL-6) trajectory class membership. Circles represent Odds ratios and solid lines represent associated 95% confidence intervals, dashed vertical line are the null value. Coefficients are adjusted for all covariates and confounders.

For probable common mental disorders (GHQ-12), being male was associated with lower odds of belonging to the high symptom class compared with females (OR = 0.73, 95% CI 0.66, 0.82) (Figure 4 and Supplementary Table 11). Older age was also protective, with decreasing odds of poor mental health trajectory observed with increasing age (≥65 years: OR = 0.57, 95% CI 0.47, 0.69). Being in a relationship compared to being single was associated with reduced odds of high mental health symptoms (OR = 0.91, 95% CI 0.83, 1.00). Furthermore, compared with White participants, those identifying as Black (OR = 0.46, 95% CI 0.36, 0.59) or Asian (OR = 0.67, 95% CI 0.56, 0.81) had lower odds of being in the worse mental health trajectory class. In terms of occupational role, doctors (OR = 0.71, 95% CI 0.59, 0.85) and non-clinical staff (OR = 0.73, 95% CI 0.66, 0.81) had reduced odds compared to nurses. A pre-existing diagnosis of PTSD (OR = 2.50, 95% CI 1.73, 3.61) or depression (OR = 4.21, 95% CI 3.67, 4.82) was associated with increased risk of worse mental health outcomes as measured by the GHQ-12.

Working in a mental health trust rather than an acute trust was associated with lower odds of being in the high mental health symptom class (GHQ-12) (OR = 0.92, 95% CI 0.84, 0.99). Redeployment was associated with decreased odds of mental health disorders (OR = 0.87, 95% CI 0.76, 0.98). Perceived support from colleagues (OR = 0.48, 95% CI 0.44, 0.54) and managers (OR = 0.38, 95% CI 0.35, 0.42) was associated with lower odds of adverse mental health trajectories.

#### Other mental health outcomes

Similar patterns of association were observed for the other outcomes. Males had lower odds of poor outcomes for probable anxiety (GAD-7) and higher odds of harmful drinking (AUDIT). Increasing age, being in a relationship, and perceiving support at work were protective across all domains. Non-White ethnic groups had lower odds of poor outcomes on most measures, although higher odds of adverse PTSD (PCL-6) trajectories. Doctors and non-clinical staff typically showed more favorable outcomes than nurses. Additionally, pre-existing mental health diagnoses were strongly predictive of poorer mental health outcomes.

## Discussion

In this national longitudinal study of clinical and non-clinical NHS staff across England (April 2020–May 2023), we found that approximately 50% of HCWs met the threshold for probable mental disorders throughout the pandemic and into the recovery phase. While symptoms of poor mental health, as measured by the GHQ-12, declined modestly early in the pandemic and then stabilized, approximately a third of HCWs experienced persistently high or worsening symptoms. The results also suggested probable anxiety and depression increased modestly from 2020 to 2021 before stabilizing, while problematic drinking and PTSD peaked in late 2020 before declining. Predictors of poorer trajectories included being female, young, single, working as a nurse, and having a pre-existing mental health diagnosis, whereas feeling supported by managers or colleagues was a consistent protective factor for depression, anxiety, and PTSD.

## Comparison with previous studies

Our findings are consistent with previous research in healthcare and other occupational groups. These studies have found persistently elevated levels of poor mental health during the COVID-19 pandemic, with similar risk factors across settings. However, NHS staff in our cohort consistently reported higher levels of distress than those observed in comparable studies, suggesting a uniquely sustained burden within this group.

Evidence from the COVID-19 Staff Wellbeing Survey, which was carried out with a smaller sample (N=585) of social care staff in Northern Ireland, found similar results for mental health outcomes over 9 months of the pandemic (Jordan et al., 2023). The study also identified two class solutions for all mental health scales, with most of the sample being classified in the ‘low-symptom’ group for depression (PHQ-9: 87% vs 84% in the ‘persistently low’ in this study), anxiety (GAD-7: 85% vs 83% in the ‘persistently low’ class in this study), and PTSD (Impact of Event Scale-Revised: 84% vs 86% the ‘persistently low’ class in this study). Similarly, the COVID-19 Staff Wellbeing Survey found that being younger and feeling unsupported were associated with membership to the high-symptom classes (Jordan et al., 2023). However, in contrast to our findings, the study found that non-clinical staff (i.e. administrative and clerical staff vs nursing and midwifery) were more likely to be in the high symptom class than nurses.

Mental health trajectories over the pandemic have also been studied in other occupational groups with comparable results. For instance, a smaller longitudinal cohort of university staff and postgraduate students found four trajectories of anxiety (GAD-7) and depression (PHQ-9) over the period April 2020–April 2021 (Carr et al., 2022). For depression, we found a comparable proportion of respondents in the ‘persistently low’ class (84% in both studies), but a higher proportion in the ‘persistently low’ class for anxiety (83% this study versus 74%, Carr and colleagues). Furthermore, similar to our findings, the study, amongst university staff and students, those who were female, younger, and had prior mental health problems had an increased risk of being in the high-symptom class.

Additionally, findings from the UK Household Longitudinal Survey (UKHLS), a UK general population study, found that mental health significantly deteriorated during the first months of the pandemic (Pierce et al., 2021). This study suggested that the prevalence of symptoms of mental health problems increased from 19% pre-pandemic to 27% in April 2020. Compared to the UK general population, NHS staff in our study showed markedly higher levels of symptoms of mental health problems during the same period. In 2020, approximately 50% of NHS staff met the threshold for probable mental disorder. As in our study, the UKHLS study found that being female and younger was associated with increased odds of adverse mental health outcomes.

Taken together, the findings suggest that being female, younger, and having a history of mental health problems were consistently associated with increased risk of poor mental health during the COVID-19 pandemic, both across different workforces and in the general population. Although similar risk factors and mental health patterns were observed across occupational groups, NHS staff appeared to experience consistently higher levels of distress throughout the study period. This pattern may reflect a persistently elevated burden of mental health problems within the NHS workforce. While some level of distress might be expected in such challenging circumstances, the consistently high and enduring levels of poor mental health among NHS staff point to more than just the impact of a temporary crisis. They highlight deeper, systemic issues within the healthcare service, such as chronic understaffing and lack of support, that have long affected staff wellbeing and were made worse by the pandemic.

## Strengths and limitations

The main strengths of this study are the large sample size and the longitudinal nature of the investigation. We included participants from 18 different acute and mental health trusts across England, which varied by rural/urban characteristics. The longitudinal cohort design enabled us to model trajectories of mental health across the acute phase of the COVID-19 pandemic and into the post-pandemic recovery phase. This has important implications for how the pandemic has impacted the mental health of HCWs in the longer term.

There are also important weaknesses. Response rates were lower for the follow-up mental health outcomes (40%, reducing to 28% for GHQ-12) compared with baseline (87% GHQ-12). We compared the baseline characteristics for people with and without complete GHQ-12 at 32-months and identified variation in completeness with age, ethnicity, clinical role, and marital status. Whether people completed their 32-month GHQ-12 did not vary by baseline outcome scores, but we do not know whether those who did not complete their baseline GHQ-12 had better or poorer baseline mental health. Completion of the mental health questionnaires may have been influenced by participants’ mental health status (i.e. people with poorer mental health may be less likely to complete questionnaires), resulting in data that are missing not at random (MNAR). However, since this study was conducted in an occupational rather than a clinical cohort, the potential impact of this source of bias on our findings is likely limited. The longer interval between the second and third follow-up waves is an additional limitation. While modeling time using the exact date of questionnaire completion allowed us to account for irregular follow-ups, this gap may have limited our ability to detect short-term changes in mental health during this period.

Furthermore, although the data covered a 3-year period, participants could only give a maximum of four responses, which limited the complexity of functional forms of the trajectories we could model. We did not include any time-varying covariates in our analyses since we were most interested in the impact of person and employment factors at baseline. Future studies may focus on how changes in personal or employment factors during the pandemic were driven by or affected the mental health of HCWs during the pandemic. Finally, while feeling supported by managers and peers was identified as a protective factor, this finding relies on self-reported data that may be subject to reporting bias. It is possible that individuals with poorer mental health were more likely to perceive or report lower levels of support. Future analyses could explore the directionality of this relationship.

## Implications for research and/or practice

The protective role of support from colleagues and managers should not be underestimated. Investing in effective peer and managerial support interventions could help improve staff wellbeing and reduce workforce attrition (Scott et al., 2025). Furthermore, given the multi-level nature of workplace support, strategies must consider how senior leadership can better equip line managers to fulfil this role.

For future pandemic preparedness, it is critical to understand why the early stages of the pandemic were especially harmful and how to respond more effectively in future crises. Additionally, although associations between support and alcohol misuse did not reach statistical significance, the effect estimates were in the expected protective direction. This may suggest the association is influenced by underreporting due to stigma or concerns about the implications of disclosing alcohol use in a professional healthcare context.

Future research should explore how broader societal pressures, such as the cost-of-living crisis and caregiving responsibilities, contribute to ongoing distress. And, considering the consistent findings that women are at increased risk of poor mental health, future research should consider this as a gendered issue which could be partly related to the fact that, on average, women are paid less and take on most caregiving responsibilities (Almeida-Meza et al., 2025; NHS England, 2025). Subsequent studies could also make better use of time-varying data and consider linking staff surveys like NHS CHECK with routine workforce monitoring to evaluate the impact of mental health initiatives over time. Furthermore, research should examine the mental health of HCWs in non-pandemic settings to establish whether the elevated symptom burden observed in the literature reflects a persistent feature of the occupation, is attributable to the crisis-related pressures, or relates more broadly to ongoing systemic pressures within the healthcare system. Methodological innovations, including tools to improve follow-up and retention, should be explored to strengthen future longitudinal research.

## Conclusions

This study provides further evidence of the substantial mental health burden experienced by HCWs during and after the COVID-19 pandemic. While many HCWs reported minimal symptoms, a substantial subgroup reported persistently poor mental health or worsening symptoms over time. Predictors of poorer trajectories included being female, younger, single, working as a nurse, or having a pre-existing mental health diagnosis. However, in keeping with research in other high-stress occupational settings, reporting feeling supported by managers and peers was found to be a protective factor, emphasizing the role of workplace culture in mitigating mental health risks. These findings highlight the need for targeted and sustained interventions to support the mental health and well-being of HCWs and improve staff retention specially during times of crisis. Such support must be ongoing and embedded within organizational practices, rather than reactive or temporary responses to specific events.

## Supporting information

10.1017/S0033291726104280.sm001 Penfold et al. supplementary material Penfold et al. supplementary material

## Supplementary material

The supplementary material for this article can be found at http://doi.org/10.1017/S0033291726104280.
