# Supplementary material for: The longitudinal trajectories of mental health outcomes in healthcare workers in England during the COVID-19 pandemic
Source: Psychol Med. 2026 May 14;56:e148. doi: 10.1017/S0033291726104280 (PMC13200146; doi:10.1017/S0033291726104280)
Supplement: Penfold et al. supplementary material [file S0033291726104280sup001.docx]

# Supplementary material

**Supplementary Table 1.** Definition of analytical sample

| NHS Check cohort | Reason for exclusion | N excluded |
| --- | --- | --- |
| **N = 24,702** |  |  |
|  | Recruited from Nightingale Trusts* | 137 |
| **24,565** |  |  |
|  | Missing weighting variables  (Trust, sex, age, ethnicity) | 1,628 |
| **22,937** |  |  |
|  | Missing job role | 173 |
| **N = 22,764** |  |  |
| *In England’s publicly funded healthcare system (the National Health Service, NHS), NHS trusts are organisations responsible for delivering healthcare services to defined populations. Each trust manages one or more hospitals and/or community services and employs healthcare workers. | | |

**Supplementary Table 2.** Guidelines for Reporting on Latent Trajectory Studies (GRoLTS) checklist

|  | **Checklist Item** | **Reported?** |
| --- | --- | --- |
| 1 | Is the metric of time used in the statistical model reported? | Yes |
| 2 | Is information presented about the mean and variance of time within a wave? | No - Completion date rather than questionnaire wave was the unit of time |
| 3a | Is the missing data mechanism reported? | Yes |
| 3b | Is a description provided of what variables are related to attrition/missing data? | Yes |
| 3c | Is a description provided of how missing data in the analyses were dealt with? | Yes |
| 4 | Is information about the distribution of the observed variables included? | Yes |
| 5 | Is the software mentioned? | Yes |
| 6a | Are alternative speciﬁcations of within-class heterogeneity considered (e.g., LGCA vs. LGMM) and clearly documented? If not, was sufﬁcient justiﬁcation provided as to eliminate certain speciﬁcations from consideration? | No. Models were specified as GMM with random effects to allow individual variability around class-specific trajectories. Alternative specifications such as LCGA assume homogeneity of trajectories within each class, which is unlikely in large occupational cohort data. |
| 6b | Are alternative speciﬁcations of the between-class differences in variance–covariance matrix structure considered and clearly documented? If not, was sufﬁcient justiﬁcation provided as to eliminate certain speciﬁcations from consideration? | No. The variance–covariance matrix was fixed across latent classes. |
| 7 | Are alternative shape/functional forms of the trajectories described? | Yes |
| 8 | If covariates have been used, can analyses still be replicated? | Yes |
| 9 | Is information reported about the number of random start values and ﬁnal iterations included? | Yes |
| 10 | Are the model comparison (and selection) tools described from a statistical perspective? | Yes |
| 11 | Are the total number of ﬁtted models reported, including a one-class solution? | Yes |
| 12 | Are the number of cases per class reported for each model (absolute sample size, or proportion)? | Yes |
| 13 | If classiﬁcation of cases in a trajectory is the goal, is entropy reported? | Yes |
| 14a | Is a plot included with the estimated mean trajectories of the ﬁnal solution? | Yes |
| 14b | Are plots included with the estimated mean trajectories for each model? | Yes |
| 14c | Is a plot included of the combination of estimated means of the ﬁnal model and the observed individual trajectories split out for each latent class? | Yes |
| 15 | Are characteristics of the ﬁnal class solution numerically described (i.e., means, SD/SE, n, CI, etc.)? | Yes - The model-estimated class-specific mean scores for the final two-class solutions are presented in Supplementary Table 10. |
| 16 | Are the syntax ﬁles available (either in the appendix, supplementary materials, or from the authors)? | Yes - All statistical code is available at DOI: https://doi.org/10.5281/zenodo.18682400 |

**Supplementary Table 3.** Comparison between people in the NHS Check cohort with complete versus incomplete GHQ-12 scores at the 32-month data collection in terms of A) their characteristics at the baseline time point, B) their primary and secondary outcome scores at the baseline time point

| **A** | | |
| --- | --- | --- |
| **Characteristics at baseline** | **32-month GHQ-12 Complete**  N (%) N = 6,329 | **32-month GHQ-12 Incomplete**  N (%) N = 16,435 |
| **Sex** |  |  |
| Female | 5,085 (80) | 13,384 (81) |
| Male | 1,244 (20) | 3,051 (19) |
| **Age** |  |  |
| ≤30 | 719 (11) | 3,895 (24) |
| 31-40 | 1,223 (19) | 3,964 (24) |
| 41-50 | 1,898 (30) | 4,040 (25) |
| 51-60 | 1,977 (31) | 3,635 (22) |
| ≥61 | 512 (8) | 901 (6) |
| **Ethnicity** |  |  |
| White | 5,683 (90) | 13,848 (84) |
| Black | 181 (3) | 800 (5) |
| Asian | 278 (4) | 1,201 (7) |
| Mixed/multiple racial or ethnic groups | 138 (2) | 432 (3) |
| Other racial or ethnic groups | 49 (0.8) | 154 (0.9) |
| **Main role** |  |  |
| Doctor | 375 (6) | 1,274 (8) |
| Nurse | 1,620 (26) | 4,249 (26) |
| Other clinical | 1,785 (28) | 5,262 (32) |
| Non-clinical | 2,549 (40) | 5,650 (34) |
| **Marital status** |  |  |
| Married/Civil partnership | 3,311 (52) | 7,737 (47) |
| Co-habiting/In a relationship | 1,413 (22) | 4,282 (26) |
| Divorced/separated/widowed | 602 (10) | 1,126 (7) |
| Single | 994 (16) | 3,249 (20) |
| Missing | 9 | 41 |
| **Type of trust** |  |  |
| Acute | 3,109 (49) | 8,148 (50) |
| Mental health | 3,220 (51) | 8,287 (50) |
| **Redeployed** (Yes) | 649 (11) | 1,987 (13) |
| Missing | 572 | 1,112 |
| **Felt supported by colleagues** |  |  |
| Not at all/moderately | 1,324 (23) | 3,549 (24) |
| Yes | 4,351 (77) | 11,411 (76) |
| Missing | 654 | 1,475 |
| **Felt supported by manager** |  |  |
| Not at all/moderately | 1,955 (34) | 5,199 (35) |
| Yes | 3,714 (66) | 9,748 (65) |
| Missing | 660 | 1,488 |
| **Pre-existing diagnosis of depression (Yes)** | 496 (9) | 1,373 (9) |
| Missing | 536 | 992 |
| **Pre-existing diagnosis of PTSD (Yes)** | 65 (1) | 200 (1) |
| Missing | 536 | 992 |
| **B** | | |
| **Baseline outcome score** | **32-month GHQ-12 Complete**  Median (Q1, Q3)  N = 6,329 | **32-month GHQ-12 Incomplete**  Median (Q1, Q3) N = 16,435 |
| GHQ-12 | 4 (1, 7) | 4 (1, 7) |
| Unknown | 870 | 2,134 |
| GAD-7 | 5 (2, 9) | 5.0 (2, 9) |
| Unknown | 2,544 | 8,764 |
| PHQ-9 | 6.0 (3, 10) | 6.0 (3, 10) |
| Unknown | 2,554 | 8,786 |
| AUDIT-C | 3 (1, 5) | 3 (1, 5) |
| Unknown | 2,722 | 9,159 |
| PCL-6 | 10 (7, 13) | 10 (8, 13) |
| Unknown | 2,547 | 8,803 |
| GHQ-12: General Health Questionnaire; GAD-7: Generalised Anxiety Disorder scale; PHQ-9: Patient Health Questionnaire; AUDIT-C: Alcohol Use Disorder Identification Test; PCL-6: Post-Traumatic Stress Disorder checklist | | |

**Supplementary Table 4.** Primary (GHQ-12) and secondary mental health outcomes (mean and caseness) from baseline, 6-month and 12-month questionnaires

| Outcome | Mean (sd) | | | | % meeting cut-off score (N) | | | | % Response rate (N) | | | |
| --- | --- | --- | --- | --- | --- | --- | --- | --- | --- | --- | --- | --- |
|  | Baseline | 6 months | 12 months | 32 months | Baseline | 6 months | 12 months | 32 months | Baseline | 6 months | 12 months | 32 months |
| Probable common mental disorders  (GHQ-12)  Cutoff≥4 | 4.5  (3.7) | 4.4  (3.9) | 4.1  (3.9) | 4.5  (3.7) | 53%  (10,450) | 51%  (4,644) | 47%  (5,051) | 50%  (3,143) | 87%  (19,760) | 40%  (9,191) | 48%  (10,845) | 28%  (6,329) |
| Probable anxiety  (GAD-7)  Cutoff≥10 | 6.3  (5.2) | 6.0  (5.4) | 5.9  (5.4) | 5.8  (5.2) | 23%  (2,592) | 22%  (2,045) | 22%  (2,311) | 21%  (1,274) | 50%  (11,456) | 40%  (9,202) | 47%  (10,735) | 27%  (6,058) |
| Probable depression  (PHQ-9)  Cutoff≥10 | 7.1  (5.7) | 6.7  (6.0) | 6.8  (6.1) | 6.6  (5.9) | 28%  (3,148) | 26%  (2,357) | 27%  (2,892) | 26%  (1,555) | 50%  (11,424) | 40%  (9,160) | 47%  (10,669) | 27%  (6,049) |
| Alcohol misuse  (AUDIT)  Cutoff≥10 | 3.8  (3.8) | 5.0  (4.1) | 3.9  (4.1) | 3.5  (4.0) | 12%  (1,329) | 20%  (1,792) | 13%  (1,426) | 11%  (682) | 46%  (10,384) | 37%  (8,445) | 44%  (10,070) | 24%  (5,377) |
| Probable PTSD  (PCL-6)  Cutoff≥14 | 11.0  (4.6) | 12.3  (5.3) | 11.2  (5.1) | 11.4  (5.4) | 24%  (2,702) | 34%  (3,211) | 26%  (2,844) | 28%  (1,694) | 48%  (10,883) | 40%  (9,003) | 47%  (10,586 | 27%  (6,067) |

**Supplementary Table 5.** General Health Questionnaire (GHQ-12) model fit indices and classification metrics

| **Functional form** | **Number of classes** | **Akaike information criterion (AIC)** | **Size adjusted Bayesian information criterion (saBIC)** | **Entropy** | **% Class 1** | **% Class 2** | **% Class 3** | **% Class 4** | **% Class 5** |
| --- | --- | --- | --- | --- | --- | --- | --- | --- | --- |
| Linear | 1 | 246638.2 | 246681.6 | 1.00000000 | 100 |  |  |  |  |
|  | 2 | 246644.2 | 246702.1 | 0.00002111 | 56.42461 | 43.57539 |  |  |  |
|  | **3** | **244441.5** | **244513.8** | **0.38557060** | **64.56911** | **0** | **35.43089** |  |  |
|  | 4 | 244447.5 | 244534.3 | 0.30079730 | 61.88006 | 0 | 0 | 38.11994 |  |
|  | 5 | 244453.5 | 244554.7 | 0.27601020 | 58.25371 | 0 | 0 | 0 | 41.74629 |
| 2 knots | 1 | 246456.5 | 246509.5 | 1.00000000 | 100 |  |  |  |  |
|  | **2** | **243887.8** | **243965** | **0.65937333** | **69.00992** | **30.99008099** |  |  |  |
|  | 3 | 246475.6 | 246576.9 | 0.00037231 | 55.88316 | 0 | 44.1168441 |  |  |
|  | 4 | 246485.6 | 246610.9 | 0.00041194 | 55.26891 | 0.009100009 | 0.7553008 | 43.966694 |  |
|  | 5 | 246495.3 | 246644.7 | 0.00053748 | 54.66375 | 0.623350623 | 0.4595505 | 0.2821003 | 43.97124 |
| 3 knots | **1** | **246422.7** | **246480.5** | **1.00000000** | **100** |  |  |  |  |
|  | **2** | **246433.8** | **246520.6** | **0.00046174** | **55.99691** | **44.00309** |  |  |  |
|  | 3 | 246446 | 246561.7 | 0.00038058 | 55.47821 | 0.05005 | 44.47174 |  |  |
|  | 4 | 246457.7 | 246602.2 | 0.00051706 | 54.94585 | 0.46865 | 0.25025 | 44.33524 |  |
|  | 5 | 246469.1 | 246642.6 | 0.00069749 | 54.6137 | 0.35945 | 0.641551 | 0.3003 | 44.08499 |

**Supplementary Table 6.** Generalised Anxiety Disorder scale (GAD-7) model fit indices and classification metrics

| **Functional form** | **Number of classes** | **Akaike information criterion (AIC)** | **Size adjusted Bayesian information criterion (saBIC)** | **Entropy** | **% Class 1** | **% Class 2** | **% Class 3** | **% Class 4** | **% Class 5** |
| --- | --- | --- | --- | --- | --- | --- | --- | --- | --- |
| Linear | **1** | **220634.8** | **220676.5** | **1.00000000** | **100** |  |  |  |  |
|  | 2 | 220640.8 | 220696.3 | 0.00003694 | 58.00432 | 41.99568 |  |  |  |
|  | 3 | 217957 | 218026.3 | 0.35259290 | 80.20378 | 0 | 19.79622 |  |  |
|  | 4 | 217963 | 218046.2 | 0.28874180 | 0 | 79.94352 | 0 | 20.05648 |  |
|  | 5 | 217969 | 218066.1 | 0.21286110 | 77.45169 | 0 | 0 | 0 | 22.54831 |
| 2 knots | 1 | 220587.8 | 220638.7 | 1.00000000 | 100 |  |  |  |  |
|  | **2** | **217658.4** | **217732.3** | **0.78634336** | **82.88388** | **17.11612** |  |  |  |
|  | 3 | 220607 | 220704.1 | 0.00045444 | 57.17925 | 1.018883 | 41.80187 |  |  |
|  | 4 | 220617 | 220737.2 | 0.00047949 | 57.29553 | 0.941359 | 0 | 41.76311 |  |
|  | 5 | 220626.6 | 220769.9 | 0.00064 | 56.476 | 0.974583 | 0.957971 | 0.509441 | 41.08201 |
| 3 knots | **1** | **220556.4** | **220611.9** | **1.00000000** | **100** |  |  |  |  |
|  | 2 | 220566.4 | 220649.6 | 0.00130512 | 60.55153 | 39.44847 |  |  |  |
|  | 3 | 220580 | 220690.9 | 0.00034637 | 58.36979 | 0.005537 | 41.62467 |  |  |
|  | 4 | 220591.8 | 220730.5 | 0.00046301 | 57.76067 | 0.271333 | 0.12736 | 41.84063 |  |
|  | 5 | 220603.4 | 220769.8 | 0.0006299771 | 58.56913 | 0.000000000 | 0.0000000 | 0.9413589 | 40.48951 |

**Supplementary Table 7.** Patient Health Questionnaire (PHQ-9) model fit indices and classification metrics

| **Functional form** | **Number of classes** | **Akaike information criterion (AIC)** | **Size adjusted Bayesian information criterion (saBIC)** | **Entropy** | **% Class 1** | **% Class 2** | **% Class 3** | **% Class 4** | **% Class 5** |
| --- | --- | --- | --- | --- | --- | --- | --- | --- | --- |
| Linear | **1** | 226414.7 | 226456.3 | 1.00000000 | 100 |  |  |  |  |
|  | **2** | 226420.7 | 226476.2 | 0.00002145 | 63.05047 | 36.94953 |  |  |  |
|  | 3 | 223457.5 | 223526.8 | 0.36044770 | 0 | 82.1797 | 17.8203 |  |  |
|  | 4 | 223463.5 | 223546.7 | 0.26339300 | 0 | 80.23295 | 0 | 19.76705 |  |
|  | 5 | 223469.5 | 223566.5 | 0.20518640 | 0 | 0 | 78.09207 | 0 | 21.90793 |
| 2 knots | **1** | **226411.2** | **226462** | **1.00000000** | **100** |  |  |  |  |
|  | **2** | **226417.7** | **226491.7** | **0.00174091** | **60.38824** | **39.61176** |  |  |  |
|  | 3 | 226430.7 | 226527.8 | 0.00031123 | 59.38991 | 0.066556 | 40.54354 |  |  |
|  | 4 | 226440.5 | 226560.7 | 0.00042334 | 60.19967 | 0 | 0 | 39.80033 |  |
|  | 5 | 226450.4 | 226593.6 | 0.00054422 | 57.24903 | 0.648918 | 1.536328 | 0.488075 | 40.07765 |
| 3 knots | 1 | 226330.2 | 226385.7 | 1.00000000 | 100 |  |  |  |  |
|  | **2** | **223086** | **223169.2** | **0.80543532** | **84.39268** | **15.60732** |  |  |  |
|  | 3 | 226353.5 | 226464.4 | 0.00042336 | 60.64892 | 0 | 39.35108 |  |  |
|  | 4 | 226365.4 | 226504.1 | 0.00051947 | 56.8386 | 3.582917 | 0 | 39.57848 |  |
|  | 5 | 226377 | 226543.4 | 0.00070412 | 59.91681 | 0 | 0 | 0.648919 | 39.43428 |

**Supplementary Table 8.** Alcohol Use Disorder Identification Test (AUDIT-C) model fit indices and classification metrics

| **Functional form** | **Number of classes** | **Akaike information criterion (AIC)** | **Size adjusted Bayesian information criterion (saBIC)** | **Entropy** | **% Class 1** | **% Class 2** | **% Class 3** | **% Class 4** | **% Class 5** |
| --- | --- | --- | --- | --- | --- | --- | --- | --- | --- |
| Linear | 1 | 189485.1 | 189526.6 | 1.00000000 | 100 |  |  |  |  |
|  | 2 | 189491.1 | 189546.4 | 0.00003892 | 63.13225 | 36.86775 |  |  |  |
|  | 3 | 184133.6 | 184202.8 | 0.55846810 | 94.3931 | 0 | 5.606901 |  |  |
|  | **4** | **181102.6** | **181185.6** | **0.91913270** | **91.22276** | **3.327172** | **4.65468** | **0.795385** |  |
|  | 5 | 184145.6 | 184242.4 | 0.28646820 | 0 | 93.72094 | 0 | 0 | 6.279057 |
| 2 knots | 1 | 188797.6 | 188848.3 | 1.00000000 | 100 |  |  |  |  |
|  | **2** | **183329.2** | **183403** | **0.94699271** | **94.65636** | **5.34364** |  |  |  |
|  | 3 | 183340 | 183436.9 | 0.46915036 | 0 | 94.30348 | 5.696522 |  |  |
|  | 4 | 188827.1 | 188947 | 0.00034537 | 60.11875 | 2.016468 | 0.005601 | 37.85918 |  |
|  | 5 | 188837 | 188980 | 0.00044750 | 60.3428 | 1.316305 | 0.229653 | 0.011203 | 38.10004 |
| 3 knots | 1 | 188837.9 | 188893.2 | 1.00000000 | 100 |  |  |  |  |
|  | **2** | **183390.8** | **183473.8** | **0.94649785** | **94.46031** | **5.539685** |  |  |  |
|  | 3 | 188856.9 | 188967.6 | 0.00138093 | 62.84658 | 0.145634 | 37.00779 |  |  |
|  | 4 | 188873.3 | 189011.7 | 0.00044570 | 60.18036 | 2.005265 | 0.089621 | 37.72475 |  |
|  | 5 | 188885.3 | 189051.3 | 0.00057904 | 59.4858 | 1.534756 | 1.758808 | 0.061614 | 37.15902 |

**Supplementary Table 9.** Post-Traumatic Stress Disorder checklist (PCL-6) model fit indices and classification metrics

| **Functional form** | **Number of classes** | **Akaike information criterion (AIC)** | **Size adjusted Bayesian information criterion (saBIC)** | **Entropy** | **% Class 1** | **% Class 2** | **% Class 3** | **% Class 4** | **% Class 5** |
| --- | --- | --- | --- | --- | --- | --- | --- | --- | --- |
| Linear | 1 | 222138.5 | 222180.2 | 1.00000000 | 100 |  |  |  |  |
|  | 2 | 222144.5 | 222200.1 | 0.00004648 | 58.83325 | 41.16675 |  |  |  |
|  | 3 | 219351.1 | 219420.5 | 0.35083320 | 84.45436 | 0 | 15.54564 |  |  |
|  | 4 | 219357.1 | 219440.4 | 0.22979250 | 81.98645 | 0 | 0 | 18.01355 |  |
|  | 5 | 219363.1 | 219460.3 | 0.18740350 | 81.42456 | 0 | 0 | 0 | 18.57544 |
| 2 knots | 1 | 221653.8 | 221704.8 | 1.00000000 | 100 |  |  |  |  |
|  | 2 | 218289.2 | 218363.3 | 0.79982142 | 86.35487 | 13.64513 |  |  |  |
|  | 3 | 221672.2 | 221769.4 | 0.00063215 | 59.99559 | 0 | 40.00441 |  |  |
|  | 4 | 221682.6 | 221803 | 0.00055199 | 59.35107 | 0.04407 | 0.027544 | 40.57732 |  |
|  | 5 | 221692.1 | 221835.6 | 0.00072474 | 57.77007 | 1.118272 | 0.60596 | 0.159753 | 40.34595 |
| 3 knots | 1 | 221951.1 | 222006.6 | 1.00000000 | 100 |  |  |  |  |
|  | **2** | **218572.8** | **218656.1** | 1.00000000 | **86.18961** | **13.81039** |  |  |  |
|  | 3 | 218584.6 | 218695.7 | 0.50796744 | 85.46246 | 0 | 14.53754 |  |  |
|  | 4 | 221978.9 | 222117.8 | 0.00190519 | 62.24866 | 0 | 0 | 37.75134 |  |
|  | 5 | 221997 | 222163.7 | 0.00098253 | 57.88024 | 1.305569 | 0 | 0 | 40.81419 |

**Supplementary Table 10.** Predicted class-specific mean outcome scores (SE and 95% CI) at each time point from the final two-class growth mixture models.

|  | **Class** | **Wave** | **Mean** | **SE** | **95% CI** | |
| --- | --- | --- | --- | --- | --- | --- |
| **GHQ-12** | Higher symptoms | Baseline | 7.76 | 0.08 | 7.62 | 7.93 |
|  |  | 6 months | 8.23 | 0.05 | 8.14 | 8.34 |
|  |  | 12 months | 7.90 | 0.08 | 7.76 | 8.04 |
|  |  | 32 months | 6.59 | 0.12 | 6.33 | 6.82 |
|  | Lower symptoms | Baseline | -0.67 | 0.07 | -0.81 | -0.52 |
|  |  | 6 months | -1.02 | 0.13 | -1.28 | -0.77 |
|  |  | 12 months | -0.04 | 0.13 | -0.31 | 0.22 |
|  |  | 32 months | 1.99 | 0.07 | 1.86 | 2.14 |
| **GAD-7** | Higher symptoms | Baseline | 12.30 | 0.22 | 11.90 | 12.70 |
|  |  | 6 months | 14.20 | 0.11 | 14.00 | 14.50 |
|  |  | 12 months | 13.90 | 0.13 | 13.70 | 14.10 |
|  |  | 32 months | 11.60 | 0.24 | 11.10 | 12.10 |
|  | Lower symptoms | Baseline | -0.18 | 0.11 | -0.39 | 0.03 |
|  |  | 6 months | -0.59 | 0.20 | -0.97 | -0.23 |
|  |  | 12 months | 0.21 | 0.20 | -0.19 | 0.56 |
|  |  | 32 months | 2.96 | 0.11 | 2.76 | 3.16 |
| **PHQ-9** | Higher symptoms | Baseline | 14.20 | 0.35 | 13.50 | 14.90 |
|  |  | 6 months | 16.60 | 0.17 | 16.30 | 17.00 |
|  |  | 12 months | 16.80 | 0.17 | 16.50 | 17.20 |
|  |  | 32 months | 14.20 | 0.35 | 13.50 | 15.00 |
|  | Lower symptoms | Baseline | -0.28 | 0.13 | -0.54 | -0.06 |
|  |  | 6 months | -1.10 | 0.21 | -1.48 | -0.73 |
|  |  | 12 months | -0.74 | 0.21 | -1.17 | -0.38 |
|  |  | 32 months | 3.06 | 0.13 | 2.79 | 3.29 |
| **AUDIT-C** | Higher symptoms | Baseline | 11.90 | 0.28 | 11.50 | 12.50 |
|  |  | 6 months | 14.30 | 0.15 | 14.00 | 14.60 |
|  |  | 12 months | 15.50 | 0.17 | 15.20 | 15.80 |
|  |  | 32 months | 16.40 | 0.28 | 15.90 | 16.90 |
|  | Lower symptoms | Baseline | 0.84 | 0.06 | 0.71 | 0.96 |
|  |  | 6 months | 0.98 | 0.11 | 0.77 | 1.18 |
|  |  | 12 months | 0.89 | 0.10 | 0.67 | 1.09 |
|  |  | 32 months | 2.81 | 0.06 | 2.70 | 2.94 |
| **PCL-6** | Higher symptoms | Baseline | 16.20 | 0.68 | 15.00 | 17.60 |
|  |  | 6 months | 20.10 | 0.12 | 19.90 | 20.40 |
|  |  | 12 months | 19.90 | 0.18 | 19.50 | 20.30 |
|  |  | 32 months | 16.10 | 0.97 | 14.10 | 18.00 |
|  | Lower symptoms | Baseline | 0.68 | 0.10 | 0.47 | 0.89 |
|  |  | 6 months | 1.29 | 0.19 | 0.93 | 1.64 |
|  |  | 12 months | 1.97 | 0.20 | 1.58 | 2.34 |
|  |  | 32 months | 3.81 | 0.10 | 3.62 | 4.00 |
| **Footnote:** Means represent model-estimated expected values for each latent class at each timepoint. Because models were not constrained to the score range, negative values may occur for low-symptom classes.  GHQ-12: General Health Questionnaire; GAD-7: Generalised Anxiety Disorder scale; PHQ-9: Patient Health Questionnaire; AUDIT-C: Alcohol Use Disorder Identification Test; PCL-6: Post-Traumatic Stress Disorder checklist | | | | | | |

**Supplementary Table 11.** Association between person and workplace factors and latent class membership

|  | GHQ-12  N = 20,089 | | GAD-7  N = 16,222 | | PHQ-9  N = 16,192 | | AUDIT  N = 16,017 | | PCL-6  N = 16,298 | |
| --- | --- | --- | --- | --- | --- | --- | --- | --- | --- | --- |
|  | ‘Persistently high’ | | ‘Moderate initial, worsening then reducing’ | | ‘Moderate initial, worsening then reducing’ | | ‘High initial, worsening’ | | ‘Moderate initial, worsening then reducing’ | |
|  | OR  (95% CI) | P | OR  (95% CI) | P | OR  (95% CI) | P | OR  (95% CI) | P | OR  (95% CI) | P |
| **Gender** (ref. Female) |  |  |  |  |  |  |  |  |  |  |
| Male | 0.73  (0.66, 0.81) | <0.001 | 0.76  (0.67, 0.90) | 0.001 | 0.99  (0.86, 1.13) | 0.85 | 2.21  (1.87, 2.61) | <0.001 | 0.99  (0.93, 1.05) | 0.77 |
| **Age** (ref. ≤30) |  |  |  |  |  |  |  |  |  |  |
| 31-40 | 1.09  (0.98, 1.22) | 0.13 | 0.79  (0.67, 0.92) | 0.003 | 0.86  (0.74, 0.99) | 0.043 | 0.96  (0.78, 1.18) | 0.70 | 0.89  (0.76, 1.04) | 0.15 |
| 41-50 | 1.01  (0.92, 1.12) | 0.76 | 0.52  (0.45, 0.61) | <0.001 | 0.62  (0.53, 0.72) | <0.001 | 1.05  (0.87, 1.26) | 0.65 | 0.71  (0.60, 0.83) | <0.001 |
| 51-60 | 0.86  (0.77, 0.97) | 0.01 | 0.38  (0.32, 0.45) | <0.001 | 0.45  (0.39, 0.52) | <0.001 | 0.84  (0.67, 1.04) | 0.11 | 0.62  (0.53, 0.73) | <0.001 |
| ≥61 | 0.57  (0.47, 0.69) | <0.001 | 0.24  (0.18, 0.32) | <0.001 | 0.27  (0.19, 0.36) | <0.001 | 0.50  (0.34, 0.74) | <0.001 | 0.48  (0.36, 0.63) | <0.001 |
| **Marital status** (ref. Single) |  |  |  |  |  |  |  |  |  |  |
| In a relationship | 0.91  (0.83, 1.00) | 0.05 | 0.89  (0.79, 1.00) | 0.05 | 0.62  (0.56, 0.69) | <0.001 | 0.71  (0.60, 0.83) | <0.001 | 0.82  (0.72, 0.92) | 0.001 |
| **Ethnicity** (ref. White) |  |  |  |  |  |  |  |  |  |  |
| Black/African/ Caribbean /Black British | 0.46  (0.36, 0.59) | <0.001 | 0.71  (0.51, 0.99) | 0.04 | 0.57  (0.39, 0.82) | 0.003 | 0.34  (0.19, 0.62) | <0.001 | 1.42  (1.07, 1.90) | 0.02 |
| Asian/Asian British | 0.67  (0.56, 0.80) | <0.001 | 0.91  (0.72, 1.15) | 0.43 | 0.96  (0.76, 1.23) | 0.76 | 0.36  (0.23, 0.58) | <0.001 | 2.10  (1.71, 2.59) | <0.001 |
| Mixed/Multiple racial and ethnic groups | 1.18  (0.91, 1.54) | 0.21 | 1.03  (0.69, 1.54) | 0.89 | 1.25  (0.90, 1.75) | 0.19 | 0.75  (0.44, 1.27) | 0.28 | 1.51  (1.10, 2.07) | 0.01 |
| Other racial and ethnic minority groups | 1.18  (0.77, 1.82) | 0.45 | 1.44  (0.83, 2.49) | 0.20 | 0.95  (0.31, 2.90) | 0.92 | 0.39  (0.12, 1.31) | 0.12 | 2.24  (1.34, 3.72) | 0.002 |
| **Main role** (ref. Nurse) |  |  |  |  |  |  |  |  |  |  |
| Other clinical | 0.90  (0.81, 1.01) | 0.06 | 0.87  (0.75, 1.00) | 0.06 | 0.86  (0.75, 0.98) | 0.03 | 0.71  (0.58, 0.86) | 0.001 | 0.73  (0.64, 0.84) | <0.001 |
| Non-clinical | 0.73  (0.66, 0.81) | <0.001 | 0.96  (0.84, 1.10) | 0.59 | 0.95  (0.83, 1.09) | 0.48 | 0.78  (0.65, 0.93) | 0.01 | 0.61  (0.54, 0.70) | <0.001 |
| Doctor | 0.71  (0.59, 0.85) | <0.001 | 0.70  (0.54, 0.89) | 0.004 | 0.53  (0.40, 0.70) | <0.001 | 0.69  (0.50, 0.97) | 0.031 | 0.42  (0.32, 0.55) | <0.001 |
| **Type of trust** (ref. Acute Trust) |  |  |  |  |  |  |  |  |  |  |
| Mental health | 0.92  (0.84, 0.99) | 0.04 | 0.68  (0.61, 0.75) | <0.001 | 0.69  (0.62, 0.78) | <0.001 | 1.22  (1.04, 1.42) | 0.01 | 0.53  (0.47, 0.59) | <0.001 |
| **Redeployed** (ref. No (usual role)) |  |  |  |  |  |  |  |  |  |  |
| Yes | 0.87  (0.76, 0.98) | 0.02 | 1.21  (1.04, 1.41) | 0.02 | 1.11  (0.94, 1.31) | 0.23 | 0.98  (0.83, 1.15) | 0.77 | 1.37  (1.17, 1.60) | <0.001 |
| **Felt supported by colleagues** (ref. No) |  |  |  |  |  |  |  |  |  |  |
| Yes | 0.48  (0.44, 0.54) | <0.001 | 0.542  (0.47, 0.62) | <0.001 | 0.49  (0.43, 0.56) | <0.001 | 0.93  (0.78, 1.13) | 0.48 | 0.53  (0.47, 0.61) | <0.001 |
| **Felt supported by manager** (ref. No) |  |  |  |  |  |  |  |  |  |  |
| Yes | 0.38  (0.34, 0.42) | <0.001 | 0.50  (0.44, 0.56) | <0.001 | 0.52  (0.46, 0.59) | <0.001 | 0.83  (0.70, 0.99) | 0.042 | 0.56  (0.49, 0.63) | <0.001 |
| **Pre-existing PTSD** (ref. No) |  |  |  |  |  |  |  |  |  |  |
| Yes | 2.50  (1.73, 3.61) | <0.001 | 4.21  (2.83, 6.26) | <0.001 | 3.46  (2.31, 5.19) | <0.001 | 1.46  (0.91, 2.32) | 0.114 | 2.49  (1.73, 3.58) | <0.001 |
| **Pre-existing depression** (ref. No) |  |  |  |  |  |  |  |  |  |  |
| Yes | 4.21  (3.67, 4.82) | <0.001 | 5.20  (4.50, 6.02) | <0.001 | 8.24  (7.11, 9.55) | <0.001 | 2.07  (1.70, 2.53) | <0.001 | 3.09  (2.65, 3.61) | <0.001 |
| **Footnote:** Coefficients are adjusted for all covariates and confounders  GHQ-12: General Health Questionnaire; GAD-7: Generalised Anxiety Disorder scale; PHQ-9: Patient Health Questionnaire; AUDIT-C: Alcohol Use Disorder Identification Test; PCL-6: Post-Traumatic Stress Disorder checklist  OR: Odds ratio; CI: confidence interval; P: p-value  PTSD: Post-traumatic stress disorder | | | | | | | | | | |


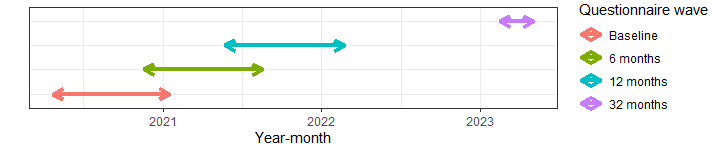


**Supplementary Figure 1.** Date of completion of baseline, 6-month, 12-month, and 32-month questionnaires
